# Editorial: AI and multi-omics for rare diseases: Challenges, advances and perspectives, Volume II

**DOI:** 10.3389/fmolb.2022.986749

**Published:** 2022-08-11

**Authors:** Silvia Bottini, Frank Emmert-Streib, Leonardo Franco

**Affiliations:** ^1^ Université Côte d’Azur, Center of Modeling, Simulation and Interactions, Nice, France; ^2^ Predictive Society and Data Analytics Lab, Faculty of Information Technology and Communication Sciences, Tampere University, Tampere, Finland; ^3^ Computer Science Department, Malaga University, Malaga, Spain

**Keywords:** rare disease, artificial intelligence, AI, multi-omics, personalized medicine, diagnosis

Gene expression is a key process in biology going from biochemical modifications of DNA to the physical arrangement of chromosomes and the activity of transcription mechanisms. Recently, several techniques have been developed to interrogate these complex processes in multiple dimensions (DNA, RNA, proteins, lipids, metabolites … ), known as “omics”. These approaches can reveal physio-pathological mechanisms in the sample. Nevertheless only the integration of several omics performed on the same sample can allow the understanding of the associated phenotype, and is essential to reveal novel insights into complex biological systems. Multi-omics data integration is an ever-increasing field at the crossroad between biology and machine learning. Nevertheless, current approaches to jointly analyze multi-omics data face multiple limitations especially when applied to rare diseases (RDs). The limited number of samples that can be collected are usually noisy, incompletely annotated, sparse, and high-dimensional (many variables), making it very challenging to develop integrative computational approaches with regard to this type of data.

RDs by definition are any disease that affects a small percentage of the population, concerning less than 1 in 2000 citizens in Europe. Although individually rare, collectively RDs are estimated to affect 350 million people globally. RDs are mainly of genetic origin and affect mainly children: 30% of children with RDs do not live to see their fifth birthday. RDs are present throughout a person’s entire life, even if symptoms do not immediately appear, and can also appear to be similar to those of common diseases. This is part of the reason for the high rate of misdiagnoses of RDs. According to the Global Genes organization, 8 out of 10 RDs are caused by a faulty gene and approximately 75% affect children, yet it takes an average of 4.8 years to arrive at an accurate diagnosis.

Given the success of volume I of this Research Topic, we have launched the second volume. We have gathered contributions that describe the current methodologies, applications, challenges facing RD diagnosis, practical insight into improving data analysis techniques as well as advances in Bioinformatics and AI approaches for biomedical research in RD. This second volume is composed of four research articles spanning multiple types of RD from alkaptonuria, an ultra-rare autosomal recessive disease to rare cancers. The multitude of approaches and applications to a wide variety of RDs demonstrate great interest in this topic.

Visibelli et al. focused on an ultra-rare disease, alkaptonuria, and developed ApreciseKUre, a multi-purpose digital platform for data collection, integration, and analysis. Employing several statistical and machine learning algorithms to analyze and re-interpret data available in the platform, they showed that patients’ stratification allows the identification of treatments that are specific to a subgroup of patients. ApreciseKUre can be extended to other diseases allowing data management, analysis, and interpretation.

Low-grade glioma (LGGs) is a rare type of cancer of the central nervous system. Although LGG is benign and good prognosis is expected, it can often transform into high-grade glioma. Therefore, there is an urgent need for the identification of LGG biomarkers for early diagnosis and the development of personalized treatment. In the work of Nguyen et al., by using the three advanced co-expression identification tools, including WGCNA, iWGCNA, and oCEM, they discovered a total of 13 hub m6A-lncRNAs. These findings can help to improve the diagnostic and prognostic power of LGG diagnosis and treatment.

Idiopathic pulmonary arterial hypertension (IPAH) is a rare disease characterized by narrowing and obliterating pulmonary vessels. Nowadays patients affected by IPAH have a poor prognosis with a low survival rate. By using protein-protein interaction (PPI) network and gene set enrichment analysis (GSEA), Yang et al. identified MAPK6 as a potential biomarker due to its discriminative power to identify patients affected by IPAH with respect to healthy patients.

Deprez et al. present a bayesian Genome-to-Phenome Sparse Regression (G2PSR), a novel multivariate regression method based on sparse SNP-gene constraints, for the joint analysis of genomics and phenomics data. They applied the G2PSR model to the Alzheimer’s Disease Neuroimaging Initiative data and relating SNPs from more than 3,500 genes to clinical and multi-variate brain volumetric information. They showed that their method can provide an accurate selection of relevant genes in the dataset with a large ratio of SNPs to samples, thus overcoming the main limitations of current genome-to-phenome association methods.

Despite the fact that multi-omics integration remains challenging especially in the field of RD, new modeling and statistical tools employing artificial intelligence methodologies are being developed to this end. We believe that this Research Topic in *Front. Mol. Biosci* (https://www.frontiersin.org/research-topics/22827/ai-and-multi-omics-for-rare-diseases-challenges-advances-and-perspectives-volume-ii) has recruited some in-depth computational approaches that dissect the fundamental principles of the role of multi-omics in RD.

